# Gliosarcoma: distinct molecular pathways and genomic alterations identified by DNA copy number/SNP microarray analysis

**DOI:** 10.1007/s11060-019-03184-1

**Published:** 2019-05-09

**Authors:** Lindsey Lowder, Jennifer Hauenstein, Ashley Woods, Hsiao-Rong Chen, Manali Rupji, Jeanne Kowalski, Jeffrey J. Olson, Debra Saxe, Matthew Schniederjan, Stewart Neill, Brent Weinberg, Soma Sengupta

**Affiliations:** 1 Department of Pathology & Laboratory Medicine, Emory University Hospital, H185D, 1364 Clifton Road, NE, Atlanta, GA 30322 USA; 2 Department of Oncology Cytogenetics, Emory University Hospital, F143A, 1364 Clifton Rd. NE, Atlanta, GA 30322 USA; 3 Department of Hematology/Oncology, Winship Cancer Institute, 1365 Clifton Rd. NE, Atlanta, GA 30322 USA; 4 Bioinformatics & Biostatistics, Winship Cancer Institute, 1365 Clifton Rd. NE, Atlanta, GA 30322 USA; 5 Department of Oncology, Dell Medical School, LIVESTRONG Cancer Institutes, The University of Texas At Austin, 1601 Trinity St., Bldg. B, Stop Z1100, Austin, TX 78712 USA; 6 Department of Neurosurgery, Winship Cancer Institute, Emory University, 1365 Clifton Rd. NE, Atlanta, GA 30322 USA; 7 Department of Oncology Cytogenetics, Emory University Hospital, 1364 Clifton Rd. NE, Atlanta, GA 143A USA; 8 Department of Pathology & Laboratory Medicine, Children’s Healthcare of Atlanta, Emory University Hospital, H185D, 1364 Clifton Road, NE, Atlanta, GA 30322 USA; 9 Department of Neuroradiology, Emory University Hospital, BG20, 1364 Clifton Road, NE, Atlanta, GA 30322 USA; 10 Departments Neurology, Hematology & Medical Oncology, Winship Cancer Institute, Emory University, 1365 Clifton Rd. NE, Atlanta, GA 30322 USA

**Keywords:** Gliosarcoma, Glioblastoma, Glioma, Oncoscan, Microarray, EGFR

## Abstract

**Purpose:**

Gliosarcoma is a histologic variant of glioblastoma (GBM), and like GBM carries a poor prognosis. Median survival is less than one (1) year with less than 5% of patients alive after 5 years. Although there is no cure, standard treatment includes surgery, radiation and chemotherapy. While very similar to GBM, gliosarcoma exhibits several distinct differences, morphologically and molecularly. Therefore, we report a comprehensive analysis of DNA copy number changes in gliosarcoma using a cytogenomic DNA copy number (CN) microarray (OncoScan^®^).

**Methods:**

Cytogenomic DNA copy number microarray (OncoScan^®^) was performed on 18 cases of gliosarcoma. MetaCore™ enrichment was applied to the array results to detect associated molecular pathways.

**Results:**

The most frequent alteration was copy number loss, comprising 57% of total copy number changes. The number of losses far exceeded the number of amplifications (***, < 0.001) and loss of heterozygosity events (***, < 0.001). Amplifications were infrequent (4.6%), particularly for *EGFR*. Chromosomes 9 and 10 had the highest number of losses; a large portion of which correlated to *CDKN2A/B* loss. Copy number gains were the second most common alteration (26.2%), with the majority occurring on chromosome 7. MetaCore™ enrichment detected notable pathways for copy number gains including: HOXA, Rho family of GTPases, and EGFR; copy number loss including: WNT, NF-kß, and CDKN2A; and copy number loss of heterozygosity including: WNT and p53.

**Conclusions:**

The pathways and copy number alterations detected in this study may represent key drivers in gliosarcoma oncogenesis and may provide a starting point toward targeted oncologic analysis with therapeutic potential.

## Introduction

Glioblastoma (GBM) is the most aggressive primary malignant adult central nervous system tumor [1, 2]. Gliosarcoma, a histologic variant, accounts for 2% of all GBMs. Overall survival of gliosarcoma is similar to GBM except that systemic metastasis and skull invasion have been reported more frequently in gliosarcoma [3]. However, gliosarcoma exhibits several unique morphologic, immunohistochemical and molecular characteristics. Gliosarcoma is defined histologically as having biphasic neoplastic components; displaying both mesenchymal (fibroblastic, osseous, muscle or adipose differentiation) and glial differentiation [2]. The genomic alterations implicated in the malignant transformation of astrocytes are diverse, of which, *IDH*, *TERT*, *EGFR*, *CDKN2A*, *TP53*, *PTEN*, *PDGFRA* and *NFKB1A* are the most commonly reported [3, 4]. These genes are involved in oxidative decarboxylation, maintenance of telomeres, stimulating protein tyrosine kinase, tumor suppression, cell signaling and a variety of other cellular processes. Gain of 7p in combination with 10q loss is associated with *EGFR* amplification and is a frequent finding in *IDH*-wildtype GBM. Of GBMs with EGFR protein overexpression, 70–90% demonstrate *EGFR* gene amplification. Other mutations such as the *EGFR*vIII and missense mutations involving the extracellular domain are distinct from *EGFR* mutations in non-glial cancers [5]. Interestingly, *EGFR* alterations vary amongst GBM subtypes, being rare in *IDH*-mutated GBM, and more prevalent in *IDH*-wildtype GBM [6]. Similar to *IDH*-wildtype GBM, gliosarcomas contain *PTEN*, *CDKN2A* and *TP53* alterations, but amplification of the *EGFR* (epidermal growth factor receptor) are uncommon (4–8%) [7–9]. Furthermore, the type of *EGFR* alterations reported in gliosarcoma are not usually seen in GBM; particularly *EGFR* point mutations have been detected in gliosarcomas (c.1831G > A) [10]. Amplification of *EGFR* is present in 35–45% of *IDH*-wildtype GBMs [2]. Of the detected *EGFR* amplifications in gliosarcoma, it is speculated that these results are derived from the glial component of the tumor rather than the sarcomatous component. Immunohistochemistry supports this, staining for EGFR is negative in the sarcomatous component and positive in the glial component [11]. Gains of chromosome 7 without *EGFR* amplification is frequent in gliosarcoma [9], leading one to believe the oncogenic driver for gliosarcomas may reside on chromosome 7, but not necessarily related to the EGFR pathway. The myriad of known candidate genes located on chromosome 7 (*CDK6, PDGF*-*A, c*-*MET*) may support this theory.

Given its poor prognosis, more research has been directed toward identifying specific mutations for targeted treatment of GBM. Several novel agents have been introduced that specifically target EGFR, but treatment with TKI’s and EGFR antibodies have not yielded successful clinical results. The hypothesis is that TKI’s work best for exon 19 and 21 mutations, which have not been detected in GBM [12]. The poor response of EGFR-targeted therapies raises the question of whether *EGFR* alterations truly represent key drivers in the genesis of glioblastomas. Therefore, we applied a whole-genome approach using the OncoScan^®^ Assay to examine DNA copy number alterations, and identify any chromosome regions known to harbor oncogenic drivers.

## Materials and methods

### Tissue processing, histopathology and clinical history review

All tissue samples were obtained for diagnostic and research (under IRB approval) purposes at the time of surgical resection. A retrospective search within the institutional pathology database for “gliosarcoma” yielded 18 cases from which a OncoScan^®^ was performed. Tissue processing consisted of fixation in 10% neutral buffered formalin and paraffin embedding (FFPE: formalin fixed paraffin embedded). Histopathologic tumor classification was rendered by multiple neuropathologists. Immunohistochemistry was performed following the manufacturers protocols using the Leica Bond Maxx III automated system for all primary antibodies: IDH1 (1:80, Dianova, Hamburg, Germany), GFAP (prediluted by manufacturer, DAKO, Carpinteria, CA). Positive and negative controls were stained alongside study materials.

Clinical data was retrieved from institutional electronic medical records and included: patient demographics, presenting symptoms, imaging characteristics, date of surgery, diagnosis, treatment, time to recurrence, length of follow-up, date of death. Less common *IDH1* and *IDH2* mutations were previously tested for clinical purposes using Emory University Hospital “SNaPshot” mutation panel, the results of which were reviewed for each case.

### Copy number (CN)/single nucleotide polymorphism (SNP) microarray analysis

For 17 specimens, formalin-fixed paraffin embedded (FFPE) brain tumor tissue was obtained and processed on Thermo Fisher’s Oncoscan SNP DNA microarray. DNA was isolated using the QIAmp DNA FFPE Tissue kit. For quantification, DNA (80 ng) was prepared with the Qubit dsDNA Broad Range assay and detected with the Qubit Fluorometer 2.0. The assay was performed according to the manufacturer’s protocol using a standard gel electrophoresis system or the Lonza FlashGel System for DNA size assessments. The assay consisted of overnight annealing of approximately 240,000 molecular inversion copy number probes and 74 somatic mutation probes, single nucleotide integration for SNP detection, two rounds of PCR, HAEIII digestion, and overnight hybridization of the libraries to the arrays. For one case, fresh tumor tissue was processed on Thermo Fisher’s CytoScan HD SNP DNA microarray. DNA was isolated with the Qiagen QIAamp DNA mini kit with the Qiagen DNA Purification from Tissues protocol. The Nanodrop ND-2000 Spectrophotometer was used to quantify the DNA (250 ng). The assay was performed according to the manufacturer’s protocol. The arrays were scanned and CEL files were processed in Thermo Fisher’s Chromosome Analysis Suite (ChAS) software: NA33 workflow and hg19 genome Refs. [13–18].

### Radiographic imaging

MRI images were manually inspected to characterize tumors according to criteria determined by the VASARI feature set [19] including: location, type of enhancement, margin definition, presence of hemorrhage or cysts, and invasion of adjacent structures such as the ependymal surfaces, pia, and cortex. The size of the T2 non-enhancing region, the T1 postcontrast enhancing region, and any areas of necrosis were recorded. Preoperative imaging (from our institution) on a comparable set of imaging from GBM patients (n = 20) from a similar time period (October 2014 and September 2015) was inspected using the same technique. Quantitative values, including measurements, were statistically compared using a two-sided *t* test assuming equal variance. Proportions between groups were compared using a Fischer exact test.

### Statistics, pathway analysis, literature review

The genes within CNV regions were obtained by using BEDTools (v2.26.0) intersect that overlapped hg19 human reference genome and CNV regions. Kaplan–Meier Survival curves were created using CASAS tool [20]. DNA copy number results were analyzed using MetaCore™ to generate molecular maps/pathways (confined to p < 0.05 for gains, losses, and LOH). Amplification events were too low in number to generate statistically significant ontology data. Literature review was attained via a Scopus^®^ search for the keywords “gliosarcoma and *EGFR*,” from 1 January 1995 to 31 July 2018.

## Results

### Clinical characteristics, radiographic features and histopathology

**Fig. 1:**
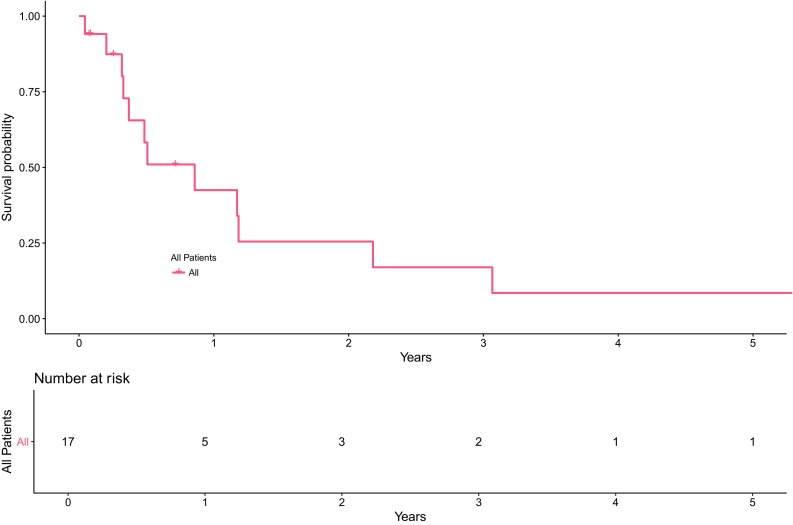
Kaplan Meier survival curves for 17 patients with gliosarcoma. The median survival was 0.9 years (313 days) (95% CI 0.3, 2.2) versus 9 months for a similar cohort of 20 *IDH*-wildtype GBM cases (9 months). Bottom graphic: number of patient’s surviving (considered to be “at risk”) after each time period

The cohort included 18 specimens (17 patients; 2 specimens were from the same patient) with a diagnosis of gliosarcoma. Twelve patients were male (70.6%), and 5 patients were female (29.4%) with a male to female ratio of 2.4:1. The median age at diagnosis was 61 years, with a range of 33–75 years. The median survival (Fig. 1) was 313 days (10.3 months) compared to 9 months (range 3–44 months) for a similar cohort of 20 *IDH*-wildtype GBM cases from our database (resected between 2014 and 2015) [21].

**Fig. 2:**
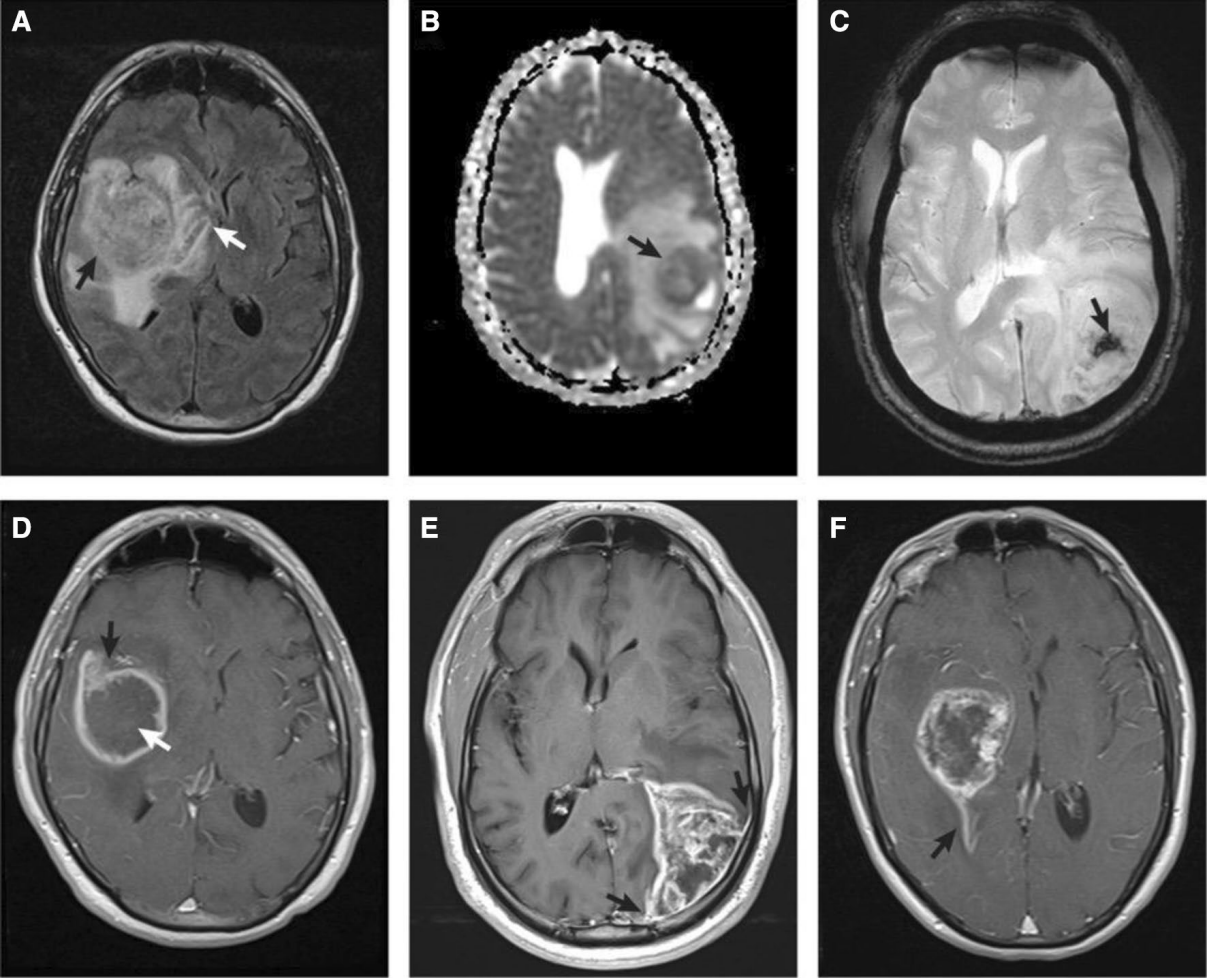
Typical MRI features of gliosarcomas. **a** FLAIR image demonstrating a centrally isointense mass (black arrow) with extensive surrounding edema extending into the basal ganglia (white arrow). **b** ADC image showing low diffusion values in the central enhancing portion of the mass (black arrow). **c** GRE image demonstrating central areas of susceptibility compatible with internal hemorrhage. **d**–**f** Postcontrast T1-weighted images showing a thick rim of enhancement (**d** black arrow) with central necrosis (**d** white arrow), pial invasion (**e** black arrows), and ependymal invasion (**f** black arrow)

Fifteen (15) patients had preoperative imaging available for review (Fig. 2). Gliosarcomas typically presented as aggressive appearing masses with extensive edema (diameter 84 mm), marked enhancement (100%), solid or thick enhancement (87%), cortical involvement (87%), pial invasion (53%), and ependymal invasion (40%). All had low or mixed average diffusion coefficient values (100%). These findings were compared to a control cohort of 20 *IDH*-wildtype GBM cases (from our institution) taken from patient’s who underwent surgical resection or biopsy between October 2014 and September 2015. The only distinct feature of gliosarcomas (compared to GBM) was a slightly larger area of edema (84 vs. 71 mm, p = 0.03 for a comparable size enhancing mass (48 vs. 43 mm, p = 0.26). Gliosarcomas were less likely to involve the occipital lobe (6% vs. 35%, p = 0.1) and have a thin rim of enhancement (13% vs. 40%, p = 0.13). Both GBM and gliosarcomas invaded adjacent structures including the cerebral cortex, ependymal surfaces, and pia, although only one (1) gliosarcoma had invasion of the adjacent calvarium, a feature which was not seen in the glioblastoma set.

All cases were diagnosed as gliosarcoma, the glial component of which, exhibited confirmatory GFAP immunohistochemical positivity. A sarcomatous morphology was present in all 18 cases, but in 3 cases the mesenchymal portion consisted of adipocytic differentiation, osteoid differentiation, and myxoid/metaplastic morphology. All cases were IDH-negative in both the glial and sarcomatous components by immunohistochemistry and *IDH*-wildtype by molecular testing. The approximate proportion of sarcomatous components in each case ranged from 10 to 60%.

### DNA copy number microarray analysis

**Fig. 3:**
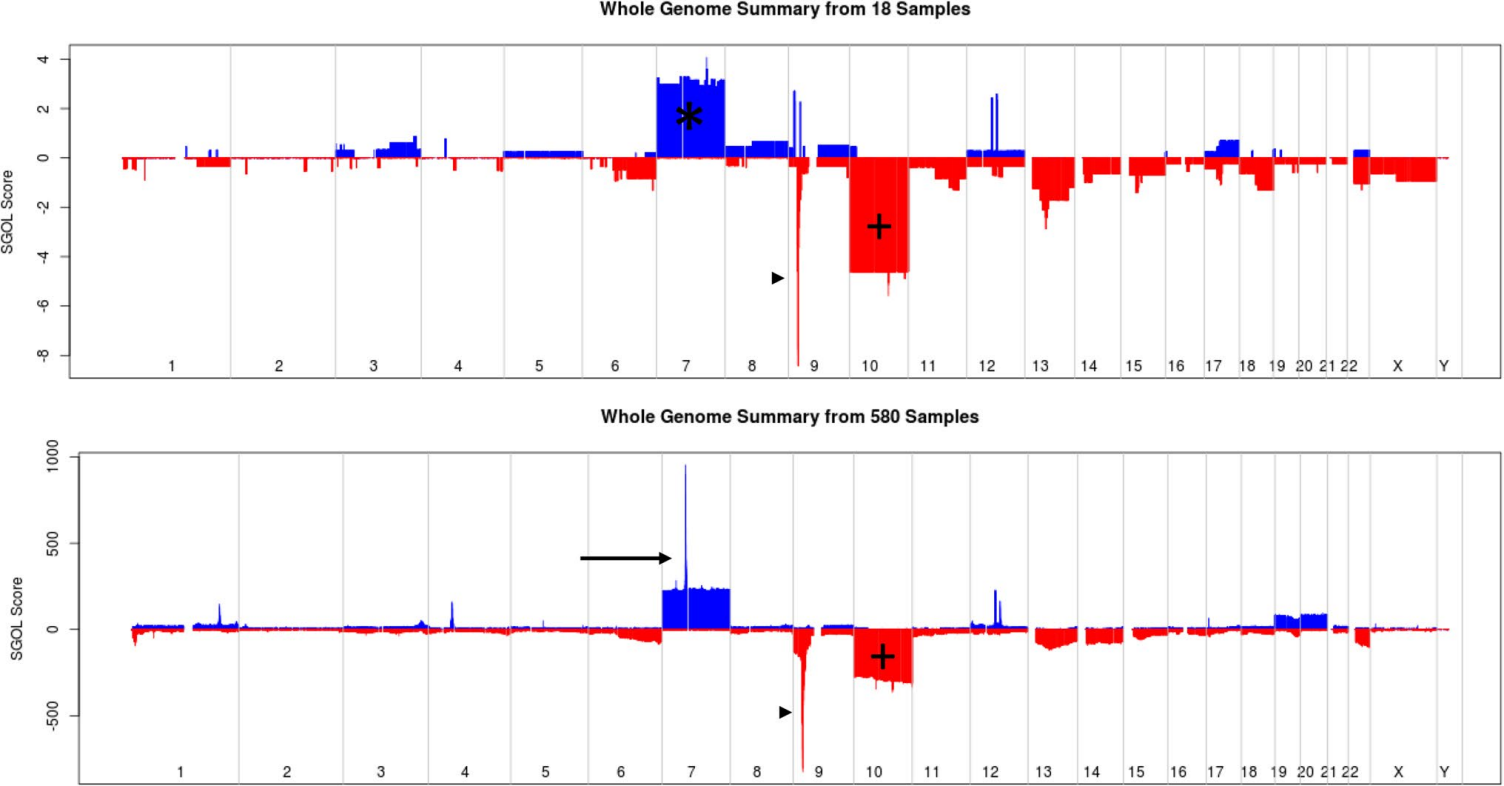
Whole genome view illustrating Oncoscan (CN/SNP DNA microarray) results. 18 gliosarcoma specimens (top) and 580 glioblastoma cases (bottom, taken from the TCGA). Focal amplification of EGFR (7p11.2) is evident in the GBM TCGA dataset (arrow), but gains of chromosome 7 (*) without EGFR (7p11.2) amplification are present in the gliosarcoma cases. Loss of chromosome 10 (+) and loss of 9p containing *CDKN2A/B* (arrowheads) are present in both GBM and gliosarcoma (+). X axis: each chromosome. Y axis: SGOL score, which represents segments of gains (blue) and losses (red) across chromosomes. Both graphics created with Copy Number Explorer. https://arraycgh.shinyapps.io/Copy_Number_Explorer/ [52]. Data from bottom graphic provided by GBM TCGA Dec 2014

Total copy number abnormalities including amplification, loss of heterozygosity, gains, and losses for 18 gliosarcoma specimens was 305 (Table 1). Per specimen, the copy number changes ranged from 6 to 52 (median = 13). Amplifications were infrequent (4.6%); were present in 6 cases and included regions on: chromosome 3p, 7q, 9p, 12q, 4q, and 7p. One case exhibited amplification of *EGFR* (7p11.2). However, gain of *EGFR* occurred in 13 cases (72%) (Fig. 3). The most frequent type of copy number alteration was loss (n = 175), comprising 57% of the total copy number changes. The number of losses far exceeded the number of amplifications (***, < 0.001) and loss of heterozygosity events (***, < 0.001). Chromosomes 9 and 10 had the highest number of losses. Particularly, chromosome 10 harbored 13 (33%) of whole chromosome losses. A large number of losses were from chromosome 9 (n = 17), mostly correlating to loss of *CDKN2A/B* (Table 1, Fig. 3). DNA copy number gains were the second most common change, totaling 80 events (26.2%). The majority of gains occurred on chromosome 7, which harbored 20 total gain events. Copy neutral loss of heterozygosity (LOH) events were infrequent, consisting of 39 events. Chromosome 17 exhibited the highest number of LOH (Table 2) in the gliosarcoma cohort. One case exhibited tetraploidy, but all other cases were referenced in diploid.

**Table 1:** Molecular alterations in 18 gliosarcoma specimens (17 patients)

Case	egfr status	Copy number abnormalities	Total CN Abn.	Amp	Gain	Loss	LOH
S13-1	Normal	1wc hmz, 1p−, 1q−, 1q+, 2p+, 3wc hmz, 3p ++(CNTN6), 3p−, 4wc hmz, 5p+, 5q−, 6wc hmz, 6p+, 7wc hmz, 7pq−, 8p−, 8q+, 9pq+, 9p−(CDKN2A/B), 9q−, 10p+, 10p ++, 10pq hmz, 10q−, 11wc hmz, 12p+, 13wc hmz, 14q+, 15wc−, 16p+, 16p−, 16pq hmz, 17p+, 17wc hmz, 18p+, 18p−, 19wc hmz, 20wc hmz, 21wc−, 22wc hmz, 22q−, Xp hmz, Xp+, Xq−	52	4	17	17	14
S14-1	Gain	1wc−, 1p+, 2wc−, 6pq cth, 6q−, 7wc+, 7q ++(MET), 9p−, 9p ++ (CER1; TEK), 9p−(CDKN2A/B), 9p+, 9pq−, 10wc−, 15wc−, 18q+, 18q ++, 18q−	18	4	3	11	0
S14-2	Gain	1q+, 2p hmz, 3p−, 6q−, 6q+, 7pq+, 7q+, 9p−(CDKN2A), 9q−, 10wc−, 11q−, 17q−, 17q+, 19q+, 22wc−	21	0	7	13	1
S15-2	Gain	6q−, 7wc+, 9p−(CDKN2A), 10wc hmz, 17q−, 22wc−	9	0	1	7	1
S15-3	Gain	1p−, 2q−, 3p hmz, 4q−, 7wc+, 9p−(CDKN2A), 10wc−, 13q−	13	0	2	10	1
S15-4	Normal	1pq+hmz, 1q−, 2wc−hmz, 3wc−, 6q−, 7pq+, 8q+, 9wc−, 10wc−, 11wc−, 12wc−, 13q−, 16wc−, 17wc−, 18wc−, 19wc−, 20wc−, Xwc−	19	0	3	16	2
S16-3	Normal	8wc+, 9p−(CDKN2A),9pq−, 10wc−, 13wc−, 14wc−, 15wc−, 17wc hmz, 18wc−, 21wc hmz	10	0	1	7	2
S16-4	Gain	7wc+, 9p−(CDKN2A), 10wc hmz, 12q−, 14q−, 15q−, 17p hmz, 19wc hmz	9	0	1	5	3
S16-5	Gain	3q−, 5wc+, 7pq+, 7q hmz, 10wc−, 11q−, 12q ++(CDK4; FRS2; MDM2), 12q−, 13q−, 16q−, 17wc+, 19q−	16	3	4	8	1
S16-6	Amp	1wc+, 2p+, 3p+, 4wc+, 4pq−, 5wc+, 6q+, 7pq+, 7wc+, 7p ++(EGFR), 8wc+, 9wc+, 10wc−, 10q−(PTEN), 13q+, 13wc−, 13q−(RB1), 14q+, 14q hmz, 15wc−, 15q−, 16p+, 16pq−, 17wc hmz, 18pq−, 20wc+, 21wc+, 22wc+	34	1	20	11	2
S17-1	Gain	This case was analyzed in reference to tetraploidy: 6q−, 7wc+, 7q ++(MET), 8p−, 9p−(CDKN2A/B), 10pq−, 11p−, 12q−, 14q−, 16p−, 17pq−−, 20q−, 22wc−	23	1	1	21	0
S17-2	Gain	1p−(FAF1), 3q+, 6p−, 7wc+, 9p−(CDKN2A/B), 9q+, 10wc−, 10q−(PTEN), 12q−, 14wc−, 17p hmz	12	0	3	8	1
S17-4	Gain	2q−, 4q−, 7pq+, 9p−(CDKN2A/B), 10wc−, 11q−, 13wc−, 15q−, 17q−	13	0	4	9	0
S18-1	Gain	2wc hmz, 2p−, 3p hmz, 3pq+, 5wc hmz, 6wc hmz, 7wc+, 8p−, 8pq hmz, 9pq+, 9p hmz, 9p−(CDKN2A), 10wc−, 12wc+, 15wc+, 16wc+, 17pq−, 17q+hmz, 18wc hmz, 19wc hmz, 20wc+, 20q−, 21q hmz	25	0	9	7	10
S18-2	Normal	3q+, 3q−, 6p−, 10pq−, 13q−, 14q−, 22wc−	10	0	1	9	0
S18-3	Gain	2p−, 7wc+, 9p−(CDKN2A/B), 10wc−, 15q−, 17wc hmz, 20q−	8	0	1	6	1
S18-4	Gain	4q++(CHIC2, PDGFRA, KIT, KDR), 7wc+, 9p−(CDKN2A), 10wc−, 15q−, 17q−	7	1	1	5	0
S18-5	Gain	7wc+, 8q−, 9p−(CDKN2A/B),10wc−	6	0	1	5	0
Total			305	14	80	175	39

Summary of copy number alterations including *EGFR* status for 18 cases of gliosarcoma. + gain, ++ amplification, − loss, − homozygous loss, *WC* whole chromosome, *HMZ* loss of heterozygosity, *CTH* chromothripsis. Manually summarized: If multiple regions of gain, loss, or amp on a chromosome, it was only represented 1 time. If it was less than whole chromosome, then pq designation. Only focal abnormalities with gene names represented

**Table 2:** Reported *EGFR* alterations in gliosarcoma

References^a^	Number of cases analyzed for *EGFR* status	Methodology^b^	*EGFR* status	Clinical outcome^c^
Reis et al. [9]	19	Differential PCR, Immunohistochemistry	No alterations (0/19)	Not available
Actor et al. [11]	38	Comparative genomic hybridization, Single-strand conformation polymorphism/heteroduplex analysis, Duplex PCR, Southern blot analysis	Amplification (3/38) Strong + IHC (2/38)^d^	Not available
Kleinschmidt-DeMasters et al. [24]	1	FISH	No amplification (0/1)	Overall survival (OS): 34 weeks
Lin et al. [28]	7	Immunohistochemistry	Strong + IHC (3/7)^e^	Median progression free survival (PFS): 0–1+ (EGFR IHC Score): 17.2 months 2–3+ (EGFR IHC Score): 11.2 months Median Overall Survival (OS): 0–1+ (EGFR IHC Score): 20.4 months 2–3+ (EGFR IHC Score): 17.7 months
Yao et al. [25]	1	FISH	No amplification (0/1)	Not available
Cachia et al. [10]	14	PCR-based primer extension assay, Next Generation sequencing, Targeted gene whole exome DNA sequencing, Immunohistochemistry	c.1831G > A (1/19)	Case #4 outcome data not denoted, not able to separate *EGFR* from WT
Shelly et al. [26]	31	FISH	Amplification (1/31)	Outcome data not stratified by histology
Pain et al. [27]	1	Next generation DNA sequencing, CISH	EGFR G719D mutation (1/1)	Not available
Smith et al. [53]	16	Not available	No amplification (0/9) No EGFRvIII mutation (0/7)	Outcome data available, not stratified by EGFR status

Summary of a literature review derived from a Scopus® search for the keywords "gliosarcoma and EGFR," from 1 January 1995 to 31 July 2018

*EGFR* epidermal growth factor receptor, *PCR* polymerase chain reaction, *IHC* immunohistochemistry, *WT* wild type, *FISH* Fluorescence in situ hybridization, *CISH* chromogenic in situ hybridization

^a^Refer to bibliography for full citation

^b^The methodology used to detect *EGFR* alterations

^c^Overall survival of cases with *EGFR* alteration versus wildtype (WT)

^d^Strong immunohistochemical staining (3+) was detected in the glial component only in 2 of the 3 cases with *EGFR* amplification

^e^Strong immunohistochemical staining (3+) was detected in the sarcomatous component in 3 of 7 cases

### Pathway enrichment (MetaCore™)

Pathways associated with copy number loss (Table 3) in the gliosarcoma cohort included WNT signaling, NF-kß, and CDKN2A. Several regions that showed chromosome loss were areas that code for WNT pathway proteins (Tcf (Lef), WNT, Sirtuin1, beta-TrCP, BMI-1, TCF7L2 (TCF4), DKK1). In addition, pathway enrichment revealed copy number loss in areas coding for components of the OX40L/OX40 pathway (NF-kB2 (p52), IKK-alpha, NF-kB2 (p100), Calcineurin A (catalytic), PKC-theta, Perforin, NF-kB). Copy number loss of the regions containing CDKN2A/p16INK4 (9p21) was present in several pathway maps. The NF-kß protein complex was also repeatedly represented.

**Table 3:** MetaCore™ pathway enrichment. Summary of proteins (“Network objects”) implicated in chromosome regions of DNA copy number gain, loss, and LOH in 18 gliosarcoma cases (17 patients)

DNA copy number type (gain, loss, LOH)		
Gain	Loss	LOH
HOXA	WNT	p53
EGFR	p16INK4/CDKN2A	Ephrin-B
Actin	NF- kß	PLD2
Adenylate cyclase	DKK1	PI3 K
PKA-reg	PTEN	MEK4
IBP3	IKK-alpha	CRK
Cytochrome C	Calcineurin A	Dsh
Rac1	MGMT	
G-protein alpha-12 family	PKC-theta	
F-actin cytoskeleton	p14ARF	
Cytochrome C		
MRLC		
IL-6		

Pathways associated with copy number gains included HOXA, EGFR, actin, adenylate cyclase, PKA-reg, IBP3, cytochrome c, Rac1, G-protein alpha-12 family, F-actin cytoskeleton, MRLC, IL-6. Gain of EGFR occurred in 13 of 18 cases (72%). Gain of regions that code for adenylate cyclase, MRLC (myosin regulatory light chains) and PKA-reg were frequent and routed to pathway maps related to myogenesis, and regulation of smooth muscle tone. The pathways with the highest statistical significance involved gains of areas containing HOXA genes, specifically centered around demethylation and methylation of histone H3 at lysine 27 (H3K27), and their role in stem cell differentiation. Rac1, a key member of the Rho family of GTPases, was a frequently identified network object related to pathways including cytoskeletal remodeling through kinase effectors of Rho GTPases. Chromosomal gains in the region coding for cytochrome c was also present, and linked to multiple maps related to apoptosis regulation and cell survival. IL-6 was frequently represented as a network object for copy number gains [22, 23].

Pathways associated with copy number loss of heterozygosity (LOH) included p53, ephrin-B, PLD2, PI3K, MEK4, CRK, and Dsh. Chromosome 17 exhibited the highest number of LOH events. This included the region encoding p53 (17p13.1), and mapped to pathways citing inhibiting of apoptosis. Interestingly, there was also WNT pathway involvement through LOH of Dsh (Dishevelled), PLD2, MEK4, and CRK; mapping to canonical WNT signaling in colorectal cancer, hepatocellular cancer, and lung cancer; frequently in the same maps associated with p53. LOH of regions encoding for several proteins related to angiogenesis was present (ephrin-B, PDF, TWEAK).

### Comparison to published data

A Scopus^®^ search for “gliosarcoma and *EGFR*,” from 1 January 1995 to 31 July 2018 yielded 58 articles, 9 of which included *EGFR* testing (Table 3). Actor et al. analyzed the largest number of cases (n = 38) and detected 3 gliosarcoma cases with *EGFR* amplification using a combination of comparative genomic hybridization, single-strand conformation polymorphism/heteroduplex analysis, duplex PCR, and southern blot analysis. Three (3) studies [24–26] used florescent in situ hybridization (FISH) to detect amplification of *EGFR*. Two (2) studies used next-generation sequencing (NGS), which detected *EGFR* mutations, c.1831G > A and G719D [10, 27]. Although EGFR immunohistochemistry was performed in 2 studies [11, 28], the ability of immunohistochemistry to differentiated *EGFR* gain from amplification is uncertain. OncoScan^®^ (DNA copy number analysis) was not performed in any of the 9 articles reviewed.

## Discussion

Overall, our data showed that DNA copy number losses were frequent and amplifications were infrequent in gliosarcoma. The majority of copy number loss occurred on chromosomes 9 and 10; localizing to regions containing *CDKN2A* and *CDKN2B* genes. The *CDKN2A* gene encodes for proteins p16 and p14arf, tumor suppressors that regulate the p53 and RB1 cell cycle [29]. The *CDKN2B* gene encodes for the p15^ink4b^ protein, a member of the p16^ink4^ (*CDKN2A*) family, and a cell growth regulator that inhibits G1 progression [30]. Homozygous loss of *CDKN2A* is common in GBM (35–50% [2]) and loss of this locus occurred in 14 of 18 gliosarcoma specimens [31]. Other potential pathways involved in gliosarcoma include activation of the OX40L/OX40 pathway, which has been shown to induce strong immunity and antitumor effects in GBM [32]. DNA copy number loss was present in areas coding for several protein components of the OX40L/OX40 pathway (NF-kB2 (p52), IKK-alpha, NF-kB2 (p100), Calcineurin A (catalytic), PKC-theta, Perforin, NF-kB). Conversely, several regions that showed chromosome loss were areas that coded for WNT pathway proteins (Tcf (Lef), WNT, Sirtuin1, beta-TrCP, BMI-1, TCF7L2 (TCF4), DKK1). In the activated or overexpressed state, most of these WNT pathway proteins (with the exception of DKK1) promote cell proliferation and cell survival [33]. However, DKK1 (Dickkopf-1) is considered to be a negative regulator of WNT pathway, and has been implicated as a candidate gene that is epigenetically silenced in medulloblastoma [34], loss of which, may provide an avenue for WNT pathway activation with subsequent cell proliferation and survival. One study showed that DKK1 expression led to glioma cell sensitivity to chemotherapy-induced apoptosis [35]. Another protein with repetitive representation after pathway enrichment was NF-kß, a protein complex that controls DNA transcription, but can induce cell proliferation and anti-apoptosis if misregulated or constitutively activated. Aberrant activation of NF-kß in glioblastoma, leading to cell invasive capabilities, resistance to radiotherapy, and even promotion of mesenchymal phenotype has been reported [36]. Studies to evaluate the therapeutic effect of inhibition of NF-kß, based on these mechanisms have been published [37]. However, due to the multifactorial role NF-kß plays in a diverse number of biological processes (cell proliferation, survival, motility, DNA repair, inflammation), a direct pathway to neoplasia in GBM is unclear. Our data shows that there is copy number loss in the region encoding for NF-kß in gliosarcoma, implying that activation of the NF-kß pathway does not play a role in gliosarcoma genesis. However, it is possible that loss of NF-kß could lead to loss of DNA repair mechanisms, resulting in neoplasia.

Chromosome gains were the second most common copy number abnormality. Gains in regions coding for signaling molecules known to promote cell proliferation (F-actin cytoskeleton, actin cytoskeletal) and cytoskeleton remodeling (G-protein alpha-12 family, F-actin cytoskeleton) were present [38]. Interestingly, the Rho GTPase family, including rac1has been implicated in the modulation of glioma cell migration through cytoskeletal rearrangement [39]. Homeobox (HOX) genes are responsible for regulation of transcription factors during embryonic development, the differential expression of which, have been tied to many different cancers, including glioblastoma [40]. Specifically, high expression of HOXA9 and HOXA10 have been reported in human glioma cell lines [40]. The expression of HOXA13 has been proposed as an activator of WNT and TGF-ß-induced epithelial to mesenchymal transition in glioma progression [41]. Resistance to radiation and chemotherapy through the activation of HOXA, thereby inducing increased proliferation and decreased apoptosis in cultured glioblastoma cells has also been reported [42]. Our microarray data showed gains in the regions encoding HOXA1, HOXA3, HOXA7, HOXA11, and HOXA13. Interestingly, several studies have demonstrated overexpression of HOX genes in Ewing sarcoma [43, 44] and undifferentiated small round blue cell sarcoma [45]; perhaps raising the question of whether HOX genes play a role in mesenchymal phenotype in gliosarcoma.

Copy neutral loss of heterozygosity (LOH) events were less frequent. OncoScan™ detected loss of the region encoding p53 (17p13.1), and while p53 alterations have been well documented in gliomas, some studies suggests that LOH of a single cell cycle regulator, such as p53 may be insufficient for development of gliomas [46]. In the context of this study, concurrent loss of p16 with LOH of p53 may play a large role in the genesis of gliosarcoma. Another pathway that was well represented across LOH, gains and losses after MetaCore™ enrichment was chemotaxis lysophosphatidic acid signaling. Lysophosphatidic acid (LPA) is a phospholipid that binds to G protein-coupled receptors (GPCRs) leading to chemotaxis, cell proliferation, cell growth and cell survival through a complex network of signaling cascades [38]. LPA was shown to be increased in GBM stem cells from the subventricular zone via LPA/Rho signaling cascades and proposed as a mechanism for GBM invasion and angiogenesis, a possible therapeutic target using LPAR antagonist and LPA synthesis inhibitors [47, 48]. Another region with copy neutral LOH encoded for the network object Ephrin-B, a protein ligand known to promote angiogenesis, as well as in developmental processes such as axon guidance, cell migration, and maturation of cortical dendrites. The Eph receptor tyrosine kinases and ephrin ligands have been implicated in both the inhibition and promotion of neoplasia and may play a key role in glioma genesis [49].

Epidermal growth factor receptor (*EGFR*) is mutated or amplified in 35–45% of *IDH*-wildtype glioblastomas [2] and implicated as a key driver [50]. Gliosarcoma, however, does not exhibit amplification or mutations of *EGFR* at the same frequency, suggesting there may be additional/alternate mechanisms propelling tumorigenesis and potentially mesenchymal transition into a sarcoma phenotype. Our data supports other studies that show a very low prevalence of *EGFR* amplification in gliosarcoma, but did show frequent gain of chromosome 7 (72%) containing *EGFR* locus. It is important to note that amplification or mutation of *EGFR* is not necessarily required for EGFR activation. For example, copy number gain of *SYNJ2* in breast cancer leads to EGFR activation by altered trafficking pathways [50]. Therefore, even though *EGFR* amplification is not common in gliosarcoma, EGFR pathway activation may still be present. Gain as oppose to amplification of *EGFR* may be sufficient for EGFR pathway activation in of itself. In contrast, some believe copy number gain (as oppose to amplification) of *EGFR* reflects chromosomal instability in cancer cells and has no biological significance [51]. Thus, it is unclear whether EGFR pathway activation is present in gliosarcoma; perhaps it is present through indirect mechanisms and not necessarily through overexpression of EGFR due to gene amplification. Our study confirms that *EGFR* amplification is uncommon in gliosarcoma and provides whole-genome evidence of possible driver pathways from a DNA copy number perspective. Further analysis to pinpoint specific loci within the altered copy number regions is needed.

The strengths of this study are that it comprises the largest cohort of gliosarcoma cases with DNA copy number analysis by OncoScan™. However, there were a few limitations. We intended this to represent an initial overview of copy number changes in a large cohort of gliosarcoma cases, and did not include OncoScan™ data from GBM. In the future, it would be ideal to compare our gliosarcoma cohort to regional-matched GBM cases. Also, no general effort was made to separate sarcomatous from glioblastoma components for OncoScan™ analysis. An interesting next step would be to separate the glioblastoma from sarcoma components microscopically, and re-analyze the separate components.

## Conclusions

Gliosarcoma, much like GBM, is a fatal diagnosis with no cure. We present a comprehensive whole-genome copy number analysis of gliosarcoma performed in an effort to identify chromosome regions that may represent plausible drivers for gliosarcoma genesis. This study may provide a starting point to direct more targeted oncologic analysis and discover genetic alterations that lead to pathways with therapeutic potential.

## Data Availability

The datasets used and/or analysed during the current study available from the corresponding author on reasonable request.

## Abbreviations

GBM: Glioblastoma
GFAP: Glial fibrillary acid protein
EGFR: Epidermal growth factor receptor
FFPE: Formalin fixed paraffin embedded
H&E: Hematoxylin & eosin
ChAS: Chromosome analysis suite
LOH: Loss of heterozygosity
Hmz: Homozygous (LOH)
WC: Whole chromosome
TCGA: The Cancer Genome Atlas
